# The Immune Epitope Database and Analysis Resource in Epitope Discovery and Synthetic Vaccine Design

**DOI:** 10.3389/fimmu.2017.00278

**Published:** 2017-03-14

**Authors:** Ward Fleri, Sinu Paul, Sandeep Kumar Dhanda, Swapnil Mahajan, Xiaojun Xu, Bjoern Peters, Alessandro Sette

**Affiliations:** ^1^Division of Vaccine Discovery, La Jolla Institute for Allergy and Immunology, La Jolla, CA, USA

**Keywords:** epitope, prediction, T cell, antibody, vaccines, MHC class I, MHC class II, immunogenicity

## Abstract

The task of epitope discovery and vaccine design is increasingly reliant on bioinformatics analytic tools and access to depositories of curated data relevant to immune reactions and specific pathogens. The Immune Epitope Database and Analysis Resource (IEDB) was indeed created to assist biomedical researchers in the development of new vaccines, diagnostics, and therapeutics. The Analysis Resource is freely available to all researchers and provides access to a variety of epitope analysis and prediction tools. The tools include validated and benchmarked methods to predict MHC class I and class II binding. The predictions from these tools can be combined with tools predicting antigen processing, TCR recognition, and B cell epitope prediction. In addition, the resource contains a variety of secondary analysis tools that allow the researcher to calculate epitope conservation, population coverage, and other relevant analytic variables. The researcher involved in vaccine design and epitope discovery will also be interested in accessing experimental published data, relevant to the specific indication of interest. The database component of the IEDB contains a vast amount of experimentally derived epitope data that can be queried through a flexible user interface. The IEDB is linked to other pathogen-specific and immunological database resources.

## Introduction

**Figure 1:**
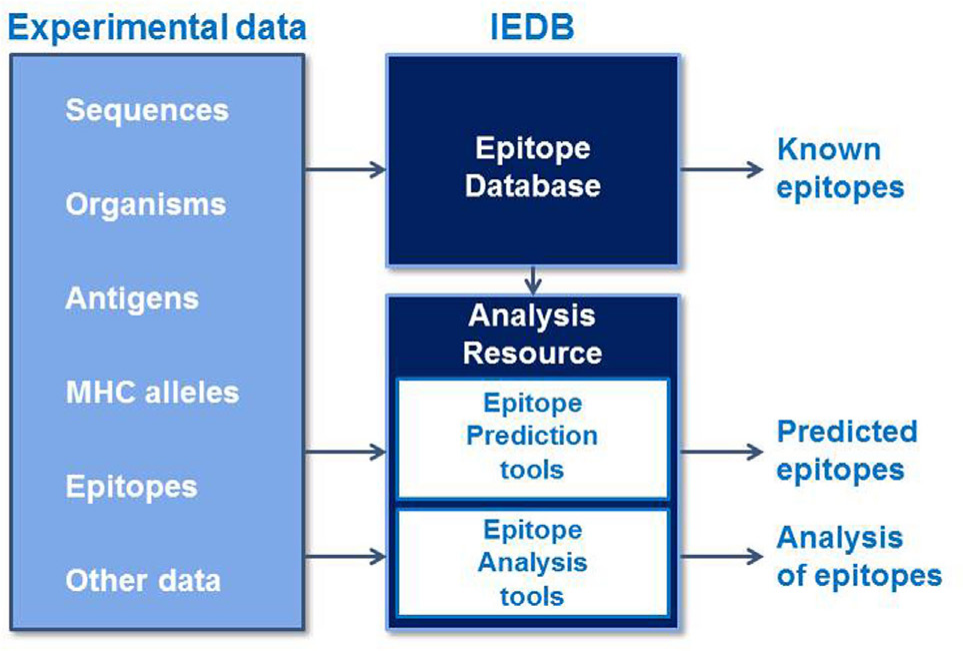
**The Immune Epitope Database and Analysis Resource captures experimental epitope data in a database and makes known epitopes freely available to the research community**. These data are used to train epitope prediction tools in the Analysis Resource, which also contains tools to analyze sets of epitopes.

The Immune Epitope Database and Analysis Resource (IEDB) is a freely available resource that contains an extensive collection of experimentally measured immune epitopes and a suite of tools for predicting and analyzing epitopes (Figure 1). The IEDB includes antibody and T cell epitopes for infectious diseases, allergens, autoimmune diseases, and transplant/alloantigens studied in humans, non-human primates, mice, and other animal species. Life science researchers can use the IEDB to develop new vaccines, diagnostics, and therapeutics. The database is populated using information captured or curated from peer-reviewed scientific literature and from data submitted by researchers. As of December 2016, over 18,000 references have been curated, and the database contains over 260,000 epitopes and over 1,200,000 B cell, T cell, MHC binding, and MHC ligand elution assays (positive and negative). Because the database is continually being updated with new literature and data submissions, the IEDB provides researchers designing vaccines with a comprehensive collection of experimental data in a single data repository that can be used to query for known epitopes and their immunogenic responses.

The tools in the Analysis Resource^1^ (1, 2) fall into two general categories: prediction and analysis tools. Prediction tools predict the outcome of experiments, such as MHC class I or class II binding, MHC class I processing and immunogenicity, and for predicting linear and discontinuous (conformational) B cell epitopes. The epitope prediction tools are valuable resources for vaccine developers when experimentally measured epitopes are not available in the IEDB. In this review, we briefly describe the basic principles of machine learning algorithms, on which the prediction tools are based, and some basic principles of tool evaluation. Next, we describe the MHC class I and class II binding prediction tools hosted in the analysis resource.

The tools for class I cover a broad range of alleles, including 83 human, 8 chimpanzee, 18 macaque, and other non-primates. The accuracy of these predictions is very high, with AUC values greater than 0.9. For MHC class II binding, the breadth of allele data is less extensive, involving 24 human alleles and 3 mouse alleles. There are also pan predictions that extrapolate from these alleles to predict binding for other alleles. The class II binding predictions are being retrained with new data as of the end of 2016. The accuracy of class II binding predictions has improved significantly over the past 10 years, from AUC of 0.76 to 0.87, but it is lower than class I. Subsequently, we describe T cell processing predictions which combine MHC binding with other parts of the MHC class I cellular pathway, namely proteasomal cleavage and TAP transport, generated from independent experimental datasets. There are also predictors trained on eluted MHC ligands that provide an overlay of the signals from MHC binding and MHC processing presentation pathway. The processing prediction tools offer a relatively small but statistically significant increase in accuracy compared to using the MHC binding prediction alone.

The Analysis Resource includes several B cell epitope prediction tools, based on a number of classical approaches such as hydrophilicity scales or amino acid properties, independently developed by different authors and reimplemented in the Analysis Resource. There are also predictors based on machine learning and structure-based approaches. The accuracy of B cell epitope prediction tools is generally rather poor, having AUC values ranging from 0.6 to 0.7. Finally, we will describe analysis tools. The analysis tools enable users to analyze known epitope sequences, assembled either from IEDB queries or other sources. These tools include epitope conservancy analysis, population coverage, and epitope clustering.

In the general categories of analysis tools, there are tools with which users can estimate the fraction of individuals expected to respond to a given set of peptides with the Population Coverage tool, calculate conservancy of a peptide within a protein, and cluster peptides based on sequence identity. The Population Coverage tool gives vaccine designers insight to the efficacy of their vaccine to regional and global populations, while the conservancy analysis tool identifies regions of a protein or antigen that are conserved and are potential targets for vaccines.

## Machine Learning and Evaluating Prediction Quality

MHC binding experiments measure the affinity between an MHC and isolated peptides, usually expressed as IC50 concentration with low IC50 value implying a high affinity binder (3). Because even a small virus can result in tens of thousands of peptide fragments as a result of processing by a cell’s MHC class I pathway, experimentalist can rarely afford to measure each of them. Machine learning approaches can develop a function that predicts affinity binding for a given peptide sequence (4, 5). Artificial neural networks (ANNs), support vector machines, linear programming, and hidden Markov models (HMMs) find this function and differ primarily in how they define “find,” their respective function spaces, and how they measure affinity binding. The calculation of a scoring matrix offers a relatively simple example. With a scoring matrix, the binding affinity for the sequence is computed based on the amino acid and its position in the binding groove. The values for each residue in the sequence are summed to yield the overall binding for the entire sequence. The position-specific scoring matrix is derived by varying the values of the matrix until the sums for known, measured peptides approximate the measured affinities. To evaluate how well this function works for predicting MHC class I peptide binding, objective methods to evaluate prediction quality are necessary.

Peptide binding datasets, far larger than any previously assembled, were originally compiled in 2006 (6). The dataset covered 48 MHC class I alleles from 88 different datasets with a variety of peptide lengths with a total of 50,000 IC50 values. This collection allowed the IEDB team to perform a thorough comparison of different prediction algorithms, including a number of publicly available prediction websites, such as SYFPEITHI (7) and BIMAS (8), and computed the correlation between the measured IC50 values and each algorithm’s predicted score, be it a heuristic score as for SYFPEITHI or a half-life of binding score as for BIMAS. To evaluate performance, given the different predicted scoring systems of the algorithms, we reformulated the problem in terms of predicting which peptides bind with an IC50 value less than 500 nM, an established threshold associated with immunogenicity for 80–90% of all epitopes.

This allowed computing the number of true negatives, true positives, false negatives, and false positives. By systematically varying the predicted score threshold from low to high, one can calculate the rate of true positives and false positives as a function of the threshold to derive an ROC curve. The area under this ROC curve is the AUC value and that has a number of important statistical properties (9). It is independent of the predicted scale because it compares the rank of your matrices and it is independent of the composition of the dataset, such as having different proportions of binders and non-binders. The AUC value is essentially capturing the probability that given two peptides, one a binder and the other a non-binder, the predicted score will be higher for the binder compared to the non-binder. An AUC value of 0.5 is equivalent to a random prediction and a value of 1.0 is equivalent to a perfect prediction.

In evaluating the different web server predictors, we discovered that it was difficult to separate the performance of the algorithms from the datasets used to train them. To correct for this effect, we used a cross-validation approach, where the dataset is split into five subsets. The algorithm is then trained on the data in four of the subsets and predicts the values in the fifth. This process is then repeated four more times, with each subset being omitted from training and used to compare predictions (10).

## MHC Class I Binding Predictions

Antigen-specific T cells do not directly recognize native antigens, but rather the T Cell receptor binds a molecular complex formed by an MHC molecule and a peptide epitope. In order for a peptide to be bound and presented by the MHC molecule, the antigen needs to be processed by the cell (11). Antigens are cleaved by the proteasome and transported into the endoplasmic reticulum (ER), through the Golgi, and finally presented in a closed groove on the MHC class I molecule. The MHC class I molecule is expressed by almost all nucleated cells and presents cleaved segments of the antigen to the CD8+ T cells. Its binding groove is closed at both ends and can accommodate peptides of 8–15 amino acids in length. MHC molecules are highly polymorphic and thousands of different variants exist. The peptide binding specificity is also very broad, and a given MHC can bind and present a number of different peptides (12).

Given the large number of variants possible and this broad specificity, experimental characterization of all peptide–MHC interactions is experimentally challenging. Binding prediction methods facilitate the selection of potential epitopes. The methods are developed using experimental peptide binding data for different MHC alleles to train machine learning algorithms that in turn can be used to predict the binding for any arbitrary peptide.

The IEDB database resource houses binding data for 173 MHC class I molecules, which includes 119 human alleles for HLA-A, B, and C. It also has data for macaque, chimpanzee, mouse, cattle, pig, and rat (13). The machine learning methods are periodically retrained when sufficient amount of new data become available in the IEDB. The prediction routines were last retrained in 2013, and the training sets are publicly available at http://tools.iedb.org/main/datasets/. The available methods and their performance have been published starting from 2005 and have seen an improvement in performance over that time (14, 15).

### The Web Interface for MHC Class I Binding Predictive Tools

The primary interface for epitope prediction tools is through a web interface, which is described below. Users can access the MHC class I binding prediction tool from the IEDB home page or directly at http://tools.iedb.org/mhci/. The class I home page has numerous tabs to assist users, including a Help tab for detailed explanations on inputs and outputs, an Example tab with specific examples and a Reference tab with publications related to the methods. From the Download tab, the user can download the scoring matrices for the various methods and a link to the dataset used for retraining the class I binding prediction tools in 2013 (16). Tool developers are encouraged to make use of these data. In addition, users can download a standalone version of the binding prediction tool that can be hosted on the user’s own server. Finally, the Contact tab has a link to the IEDB help desk at help@iedb.org that has the goal of responding within one business day of receiving a help request.

To perform class I binding predictions, the user inputs one or more sequences as plain text, separating the sequences with blanks, or in FASTA format, or specifies a file containing the proteins. Next, a user can select a preferred method from a list including IEDB recommended, consensus (17), netMHCpan (10, 18), ANN (4, 19–22), scoring matrix method (SMM) (5), SMMPMBEC (23), Combinatorial Library (24), PickPocket (25), and netMHCcons (26). The IEDB recommended method is the default setting and usually is consensus, a combination of three different methods (ANN, SMM, and Combinatorial Library). If these methods are not available for the selected allele, netMHCpan will be used instead. The user next specifies MHC species (human, mouse, non-human primates, and others) and specific alleles. Multiple alleles and epitope lengths (9–14) can be specified. For humans, the most frequent alleles are available for selection by default, and a reference set of the 27 most frequent alleles (97% of the global population) and peptide lengths of 9 and 10 can also be selected.

### Output of MHC Class I Binding Predictive Tools

When the user clicks the submit button, the protein sequence is parsed into all possible peptides for the specified length and the predicted binding affinity for each is calculated. The tool compares the predicted affinity to that of a large set of randomly selected peptides and assigns a percentile rank (lower percentile rank corresponds to higher binding affinity), which is method independent since not all methods predict IC50 values. In the Consensus method (17), the median value of the three values is used. Results are presented by default sorted by predicted percentile rank, but results can also be sorted by sequence position. Users can filter results by a designated percentile rank cutoff.

The results page lists the input protein sequence, the table of results, a link to download the results as a CSV file, and a list of citations associated with the methods used. The output table includes columns for the allele, peptide start and end positions, the peptide length, the peptide sequence, the method(s) used, and the percentile rank. Checking a box at the top of the results table can expand the results. The output table can be sorted by clicking on each column header.

There are three main strategies for selecting potential binders. The first involves selecting all peptides with IC50 value less than 500 nM, a threshold previously associated with immunogenicity (3). A second recommended strategy is to pick the top 1% of peptides for each allele/length combination. The third strategy is to pick peptides with percentile ranks below 1% (27). A further study regarding the strength of affinity for different alleles and their repertoire size indicated that repertoire size differs for each allele (28). We derived from this study allele-specific binding thresholds for the 38 most common HLA-A and HLA-B alleles. A link at the top of the Help tab accesses a table of alleles and their associated affinity cutoff IC50 values.

### Standalone Version and Application Program Interface (API)

The MHC class I binding tools are also available in a standalone version that runs on a Linux operating system and can be downloaded from the Download tab. Because the standalone version is hosted locally on hardware of the user’s choosing, an internet connection is not needed and users do not need to worry about their web browser timing out. Instructions for installing the software are provided in the accompanying README file.

**Figure 2:**
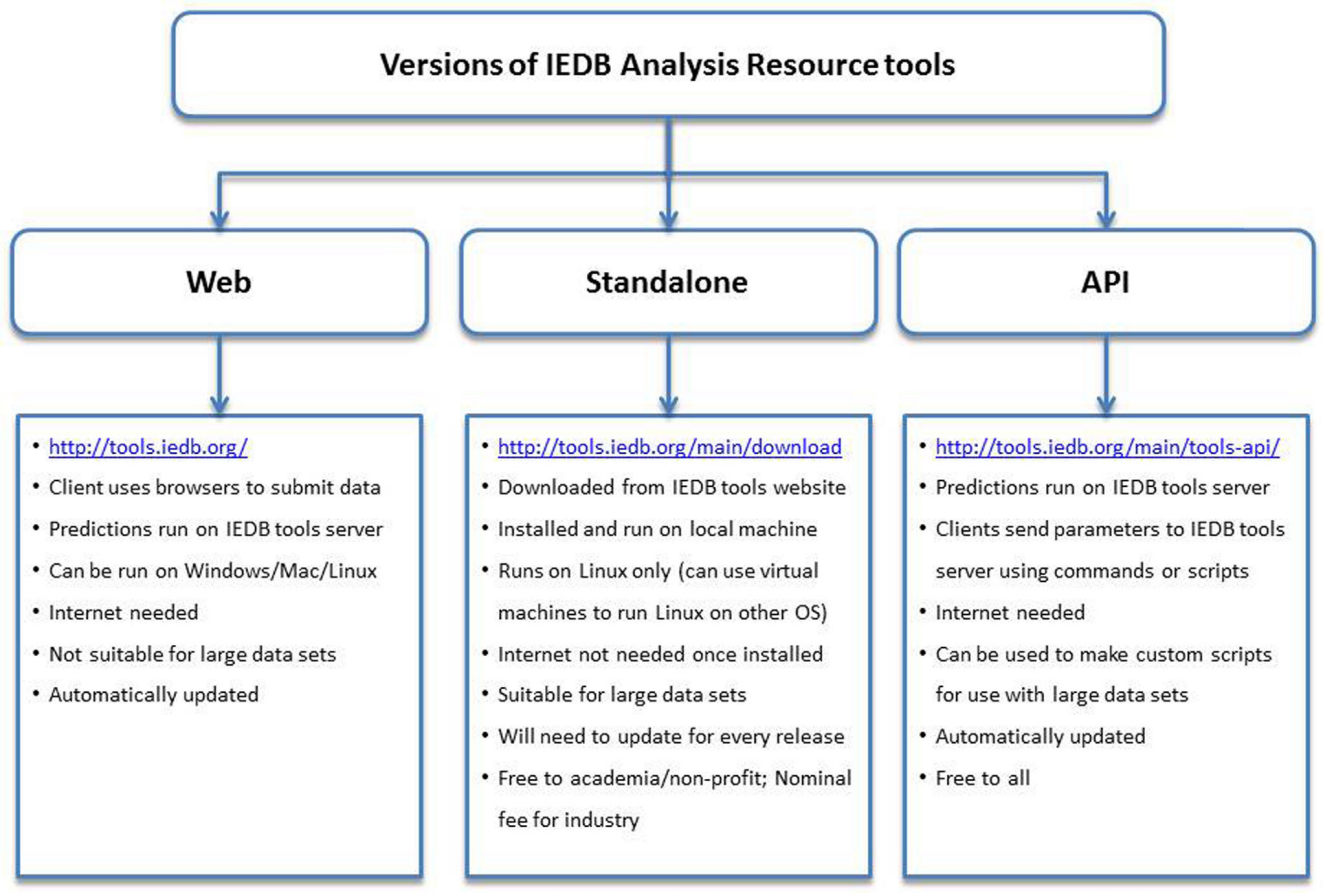
**Different versions of the prediction tools available at Immune Epitope Database and Analysis Resource (IEDB) and their features**. The web version has an easy to use interface and can be accessed using internet browsers at http://tools.iedb.org/. The standalone version can be downloaded from the IEDB tools website and installed on local computers. The Application Program Interface (API) version can be used to make custom scripts and used in pipelines.

All users can also access the MHC binding prediction tools using the API. The API uses a Representational State Transfer, an application that uses HTTP requests to GET, PUT, POST, or DELETE data from web servers. In this manner, users can send parameters over the internet directly to the IEDB web servers. No special software needs to be installed locally to use the API, and users will always access the latest version of the tools, unlike the standalone versions that need to be updated on local servers with each new release of the prediction tools. The API can also be incorporated into scripts and pipelines. In addition to the MHC class I binding prediction tool, APIs exist for several other prediction tools, including class II binding, linear antibody epitope, class I processing, class I immunogenicity, and MHC-NP. Documentation for using these APIs can be found on the Tool-API tab.^2^ The features and attributes of the web, standalone, and API versions of the tools are summarized in Figure 2.

## MHC Class I Processing Prediction Tools

When a virus infects a host, it gets inside the cell, replicates, and spreads throughout the host. The virus and other components are degraded by a process known as antigen processing, which results in some of the peptide fragments being presented on the surface in the context of MHC class I epitopes that are recognized by CD8 T cells that can kill the cell, produce cytokines, proliferate, and form memory populations. Antigen processing is a complex enzymatic process with key players such as the proteasome complex that generates short peptides. Some of these fragments are transported from the cytosol into the ER by binding to TAP, where they undergo further trimming of N-terminal residues and then bind to MHC complexes. These are then transported to the cell surface where they can be presented to T cells (11).

To predict the outcome of antigen processing and presentation, proteasomal cleavage, TAP transport, MHC class I binding, and T cell receptor peptide–MHC interaction can be considered. Each step of this pathway has a corresponding specificity or efficacy. As a first approximation, about 15% of all peptides that can be made from a protein are actually transported into the ER and about 2.5% of peptides that are made will bind to an MHC molecule. About 50% of peptides presented on the cell surface will be recognized by a T cell receptor (29).

**Figure 3:**
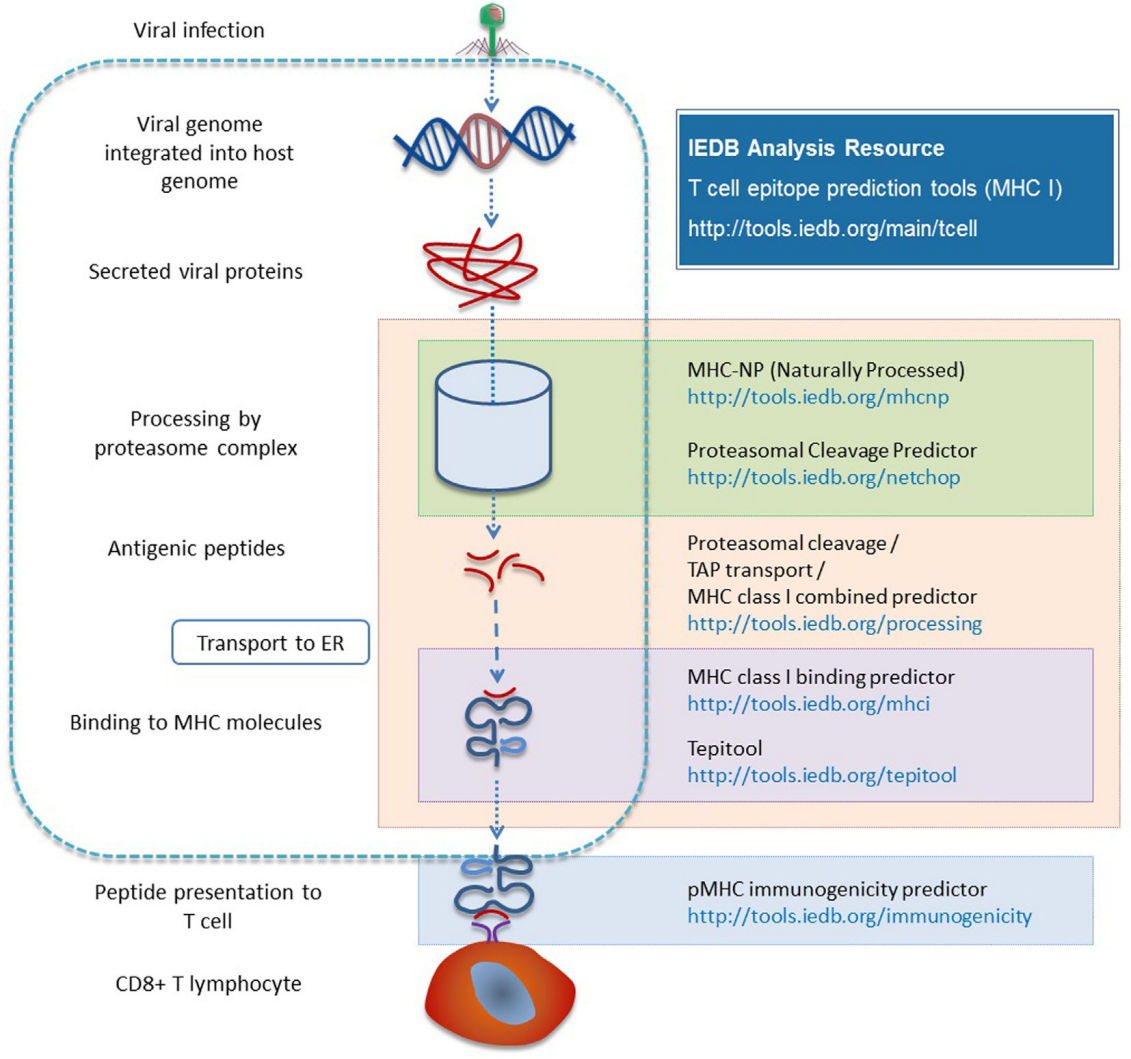
**Different prediction tools are available in the Analysis Resource with respect to different stages of MHC I antigen processing**.

There are two different types of processing tools in the Analysis Resource. One combines proteasomal cleavage, the transfer of peptide fragments by TAP, and MHC binding. The other type is neural network trained directly on naturally processed and presented peptides. The NetChop method (30, 31) models the cleavage and generation of the C-terminus of peptides based on data from naturally processed peptides and it is combined with other neural network based approaches [NetCTL (32) and NetCTLpan (33)]. Processing prediction tools include the MHC-NP tool developed by a research group outside the IEDB team, based on MHC elution experiments to assess the probability that a given peptide is naturally processed and binds to a given MHC molecule (34). Figure 3 provides a summary of the various tools involved in antigen processing and the different steps of processing with which they are associated.

### Combined Predictors

This tool can be found on the T Cell Tools section of the web interface, similar to that of the MHC class I binding, with added sections for proteasome cleavage and TAP transport. Users can select between two proteasome types—immunoproteasome and constitutive proteasome. Since their specificities are very similar (35), the immunoproteasome is recommended for use and is set by default.

TAP transports peptides into the ER that are potentially N-terminally extended from the ligand that end up in MHC (36). This means that the peptide that binds to MHC does not necessarily need to be a good substrate for TAP, but needs to be an N-terminally extended precursor of a TAP substrate. Accordingly, the TAP transport input section has two input fields, maximum precursor extension and alpha factor. The TAP transport method takes a precursor of up to an extension of 1 into account. The alpha factor is a weighting factor that takes into account the uncertainty of not knowing which peptides are involved. A detailed discussion on the selection of these values has been published elsewhere (37). For most users, we recommend the use of the default values of 1 for the maximum precursor extension and 0.2 for the alpha factor as these worked best in predicting TAP transport overall.

The output includes columns for the allele, start and end positions, peptide length, and sequence, plus columns for proteasome, TAP, MHC, and Total scores. The proteasome score indicates how well the peptide with its C-terminus could have been generated. The TAP transport score evaluated the ability of the peptide or its N-terminally prolonged precursor to be generated. The MHC score is the −log10 of the IC50 value, so in this case a higher MHC score indicates a better binder. The total score is the sum of these three scores.

There are several caveats associated with the combined predictor and the other processing prediction tools. Based on our evaluation of the performance of prediction methods, we found that the processing predictions were better than the MHC binding predictions alone when predicting eluted peptides (38). However, eluted peptides are typically identified by mass spectrometry, which requires the peptide to be reasonably abundant to allow for detection. In actuality, an immune response could occur with few peptides on a cell. So there is a potential bias that eluted data overrepresents the “best possible” ligands and the difference between the processing predictions and the binding-only predictions may not be relevant in practice. When we benchmarked the ability to predict T cell epitopes, the improvement was not statistically significant. In conclusion, while processing predictions make sense in terms of the biology, the IEDB team recommends using the MHC class I binding predictions, which are trained on much larger datasets. For situations where the binding predictions provide too many candidate epitopes, using the processing scores instead can offer another filter to reduce the number of peptides to investigate.

### Other Processing Tools

Additional processing predictors are the neural network based tools, NetChop (30, 31), NetCTL (32), and NetCTLpan (33). NetChop is the proteasomal cleavage predictor based on an analysis of C-terminus residues in eluted ligands. NetCTL uses the NetMHC method and combines it with NetChop and TAP transport method. NetCTLpan uses the NetChop, TAP transport method, and NetMHCpan.

The input interface is again similar to the other binding and processing tools, allowing the user to specify one of the three prediction methods and the protein sequence(s) of interest. NetChop predicts C-terminal cleavage based on two approaches, either the C-term 3.0 method, which uses specificities for the C-terminals based on eluted MHC ligands, or the 20S 3.0 method, which uses the analysis of proteasomal cleavage digests similar to the combined predictor. C-term 3.0 is not actually a proteasome prediction because it derives the specificity of the C-terminals statistically from eluted ligands that reflect the TAP transport specificity, but it performs better than 20S 3.0 and is, therefore, presented as the default value for all three methods. There are six publications that provide further details on the methods.

An additional processing method is MHC-NP, which was contributed to the IEDB by the Giguère group. While it covers a limited number of mouse and human MHC class I alleles, MHC-NP won a benchmark performance contest at the Second Machine learning Competition in Immunology.^3^ The IEDB is open to hosting tools from external groups on the Analysis Resource and welcomes the opportunity to do so.

## The T Cell Immunogenicity Predictor

The Analysis Resource has a T cell immunogenicity predictor tool that predicts the relative ability of a given set of peptides bound in an MHC complex to be recognized by a T cell. To develop the tool, we assembled datasets of peptides that have similar MHC binding affinity and then separated the ones that are recognized by T cells from the ones that are not recognized by T cells. We then observed that certain amino acids, such as tryptophan, phenylalanine, and isoleucine, are enriched in immunogenic peptides while other residues, such as serine, methylamine, and lysine, are depleted. Studies suggest that tryptophan and phenylalanine have long side chains that have a greater ability to make contact with the T cell receptor, possibly making them more immunogenic than other residues (39). We used the enrichment and depletions score to generate propensity scales than can be used to evaluate a peptide directly (39).

The tool has only been validated for 9-mers. By default, the tool masks the first, second, and C-terminus residues, the ones most likely to be directly responsible for MHC binding. The remaining residues in positions 3–8 are then the ones most likely to be in contact with the T cell and they are evaluated using the propensity scale and a score is calculated. A positive score indicates a likelihood of T cell recognition and a negative score indicates that recognition is less likely. We have conducted extensive studies to validate this approach. The tool has AUC values of 0.65–0.69, which is rather poor but still statistically significant. It offers an advantage over the processing tools in that it is independent of MHC binding.

It is worth noting that proteasomal cleavage, TAP transport, and MHC binding have undergone coevolution to a large extent, so that MHC molecules have evolved to bind peptides that are in the ER (38). So processing prediction tools are predicting dependent variables. As a result, their combination does not provide vastly improved predictions. In contrast, the immunogenicity tool focuses on residues that are not involved in these other predictions, and thus, its results are independent of the other processing prediction tools. Therefore, the IEDB team recommends using the MHC class I binding predictions to select candidate peptides for measurement and supplement that with the immunogenicity predictor to further reduce candidates to test.

## MHC Class II Binding Predictions

The MHC class II antigen-processing pathway applies to exogenous proteins from extracellular sources, such as bacteria or fungus, which are engulfed by the cell and cleaved by proteases in the lysosome (11). The MHC class II molecules are synthesized by the ER and have two chains, alpha and beta, which assemble together to make a complete MHC class II chain. After a series of complex cellular processing events, MHC class II carrying specific peptides derived from degradation of these proteins are presented on the cell surface to T cell scrutiny (11).

The basic structure and principles for class II and class I binding prediction have many things in common but there are also some important differences (Table 1). With regard to structure, MHC class I molecules have a single alpha chain that impacts binding and the binding cleft lies between the alpha 1 and alpha 2 domains. Because the binding groove is closed, it can only accommodate shorter peptides (8–14 amino acids). MHC class II molecules though have two chains, alpha and beta that impact binding (12). The binding groove is open and can accommodate longer peptides (13–25 amino acids). MHC class I molecules are present in all nucleated cells. Class II molecules are found only in antigen presenting cells, such as macrophage cells, B cells, or dendritic cells (12).

**Table 1:** **Comparison of MHC class I and class II epitope prediction tools available in the IEDB**.

Features	MHC class I	MHC class II
Structure	3 Alpha chains, 1 beta-2 microglobulin	2 Alpha chains, 2 beta chains
Peptide binding chain	Alpha	Alpha and beta
Binding cleft	Closed	Open
Loci	A, B, and C	DP, DQ, and DR
Antigen presenting cells	All nucleated cells	Dendritic, macrophage and B cells
Responding lymphocytes	Cytotoxic T cells (CD8+)	T helper cells (CD4+)
URL for IEDB predictions	http://tools.iedb.org/mhci	http://tools.iedb.org/mhcii
Binding core	9 residues	9 residues
Residues flanking the binding core	NA	0–5 on each side
Recommended cutoff (IEDB Consensus percentile rank)	1.0	10.0
Peptide length accepted	8–14-mer	15-mer
Algorithms available	8 (Consensus, NetMHCpan, artificial neural network, SMMPMBEC, SMM, Comblib, PickPocket and NetMHCcons)	6 (Consensus, NetMHCIIpan, NN-align, SMM-align, Combinatorial library, Sturniolo)
Host species for which prediction is available	8 (human, mouse, gorilla, chimpanzee, cow, macaque, pig, and rat)	2 (human and mouse)
Total number of alleles available	3,600	DPA = 17, DPB = 128, DQA = 28, DQB = 104, DR = 256

*IEDB, Immune Epitope Database and Analysis Resource; SMM, scoring matrix method*.

With regard to nomenclature (40), only the alpha chain is variable in class I molecules so the nomenclature is “HLA” followed by the locus, typically A, B, or C, an asterisk, and certain digits that define the kind of allele it represents. An example for class I is HLA-B*07:02. For class II molecules, both the alpha and beta chains affect binding and both chains are variable for the DP and DQ loci. As such, both chains need to be specified, such as HLA-DPA1*01:03/DPB1*02:01. For the DR locus, only the beta chain is variable so it is the only one that needs to be specified, for example HLA-DRB1*01:01.

Another important difference pertains to the binding core. Because the open binding groove accommodates longer peptides, only part of the peptide binds or interacts with the class II molecule. The binding core is typically nine amino acids in length with neighboring or flanking residues. As a result, it is difficult to identify which residues are actually involved in the binding process. For proper binding, the binding core needs to be aligned with the binding groove. Flanking residues also interact with the class II molecule outside the binding groove, but because peptides lengths typically vary from 15 to 23 residues, the flanking residues are also challenging to identify. As a result, MHC class II binding prediction is more challenging than that for class I molecules (41–45).

### MHC Class II Binding Prediction Tool Web Version

The web interface for the MHC class II binding prediction tool has many similarities to the class I interface. The tool can be accessed by links on the IEDB home page or directly at http://tools.iedb.org/mhcii and has tabs for Help, Example, Reference, Download, and Contact. Users can find a sequence of interest by clicking on the NCBI sequence browser and cut/paste it into the sequence field. The format for the sequences can be plain text or FASTA. Users can also upload a file with their sequences as plain text.

Several different prediction methods are available including IEDB recommended, Consensus (41, 46), netMHCIIpan (47, 48), SMM-align (49), Sturniolo (50), and NN-align (51). The default method is IEDB recommended. Users can specify the species and locus for humans (DR, DP, and DQ) and mouse (H-2-I), and the associated alleles. Finally, users can specify the output results to be sorted by percentile rank or sequence position. The IEDB team continuously evaluates and enhances algorithms and develops new algorithms, so the IEDB recommended methods can change over time. Each method generates a percentile rank and a binding affinity score.

The user selects the species, locus, and alleles. As in the class I binding tool, only the most frequent alleles are listed for HLA-DR. There are five or six alleles available for the DP and DQ loci. Thus, there are fewer alleles available, because less data are available for training the algorithms (44). By default, the alpha and beta chains are combined. If the user wants to specify the chains separately for DP and DQ, they can check a box to enable this option. For the DR locus, only the beta chain is displayed since the alpha chain is invariant.

A reference set of 27 most common alleles (52) that can provide global coverage can be selected by checking the appropriate box. Users also have the option to upload allele selections in a text file using the allele names. The tool parses the input protein sequences into 15-mers and predicts the binding affinity for each peptide. It then compares the predicted affinity for each peptide with that of a large set of randomly selected peptides to determine its percentile rank. The Consensus method uses the median rank of the three constitutive methods. As before, the lower the percentile rank, the better the binder.

Users can download the results in a CSV file and the web output lists the allele in the first column, followed by the start and end sequence positions, the peptide sequence, method used, and the percentile rank. The results can be expanded by checking the box at the top of the table, which reveals the different scores for the methods used. The expanded view also lists the nine amino acid binding core computed by each method.

As for class I, there are several recommended approaches to selecting binders, including selecting peptides that have percentile rank less than or equal to 10.0, or IC50 values less than or equal to 1,000 nM, the experimentally determined threshold for class II immunogenicity (53). Another approach is to select a desired percentage of the peptides within the peptide set sorted by percentile rank for users who want to study a fixed number of best possible peptides.

Because the tool breaks down input sequences into all possible 15-mers, many of these sequences will have the same predicted 9-mer binding core. One way to reduce this redundancy is to preprocess the input protein sequence by splitting it into a series of 15-mers that overlap by 10 residues and submit this series as input instead of the entire protein sequence. An overlap of 10 is recommended because it can capture the minimal number of 15-mers with all possible 9-mer binding cores with at least on flanking residue on both sides. Alternatively, the user can post-process results when the entire protein sequence is used as input by selecting the best binding (lowest percentile score) peptide among those with the same binding core. Because this process is more involved than the pre-processing approach, the pre-processing approach is recommended.

### Prediction of Promiscuous Binders and Immunodominant Epitopes

A peptide that binds to multiple MHC molecules is referred to as a promiscuous binder. Promiscuous binders may be associated with strong antigenicity (54, 55) and can provide extensive population coverage (52, 55). To predict promiscuous class II binders, the binding prediction tool can be used as described but raising the binding percentile rank threshold to 20 from 10, opting to use the 27 reference alleles that covers 94% of the global population. Once the run is submitted and completed, users can download the results into a CSV formatted file and use a spreadsheet program to find the sequences that have percentile rank below 20, and then count the number of alleles that bind each 15-mer peptide. We recommend selecting sequences that bind at least 50% of the alleles with a percentile rank cutoff of 20%.

In an independent study, we analyzed peptide datasets with measured immune responses from house dust mite, Timothy grass, *Mycobacterium tuberculosis*, cockroach allergens, and Pertussis (44). The aim of the study was to devise prediction strategies not at the level of single alleles, but rather at the level of the general population. After extensive experimentation, we discovered that a combined prediction for a set of seven alleles, representative of prototypic binding supertypes, could capture 50% immune response with 20% of the peptides. Users can generate 15-mers overlapping by 10 residues from their protein of interest, predict binding for these seven alleles, compute the median consensus percentile rank of the seven values for each peptide, and select all peptides with a median consensus percentile rank less than or equal to 20.0. This group of peptides will capture ~50% of the immune response in a general human population (44).

## TepiTool

The IEDB team has recently developed TepiTool, a T cell Epitope Tool that provides a user-friendly interface for MHC class I and II binding predictions, by using IEDB team’s recommendations as defaults, to automatically select the top peptides (56). The TepiTool interface is a step-by-step wizard that guides the user through input parameters and desired outputs. It is currently available at http://tools.iedb.org/tepitool on the Analysis Resource Labs web page.

TepiTool guides the user through six separate steps, specifying inputs about their sequences (step 1), host species and MHC class (step 2), and alleles (step 3). For class I alleles, there are several options that the user can pick via radio buttons. The user can select from the list of frequently occurring alleles (frequency greater than 1% in the global population), a list of all available alleles, a list of representative alleles from different HLA supertypes, the panel of 27 allele reference set, or they can choose to upload an allele file. In step 4, the user is presented with several options regarding the selection of peptides, including low, moderate, or high number of peptides and different lengths of peptides. The user also has the option to select their own settings regarding removing or keeping duplicate peptides (to facilitate setting up of pools for screening). The user also has the option of including only peptides that are conserved in a specified percentage of sequences. By default, the percentage is set to 50%, but it can be changed in 10% increments up or down. All during this process, the selected parameters are summarized in a panel on the right of the web page.

In step 5, the user selects the prediction method (IEDB recommended, Consensus, SMM, or ANN) and the output (predicted percentile rank, predicted IC50 or MHC-specific binding thresholds, the top X% or the top X number of peptides based on percentile rank). The recommended threshold of 500 nM is provided as a default if the IC50 option is chosen. In all cases, the number of peptides in the predicted results is shown in the summary panel on the right. In the sixth and final step, the user reviews input parameters and selected options before submission. The user can also specify a job name and an email address to be notified when the run is completed.

On the output page, a table of concise results and the best binders based on the chosen criteria are listed at the top. This table contains the peptide start and end positions, peptide sequence, percentile rank, allele name, and the level of conservancy (if this option was chosen). The output also contains links to download the complete results citation information for the tools used, the input sequence, and a summary of the other input parameters.

MHC class II binding predictions are performed similarly, and in step 3, the user can select custom allele sets, the seven-allele, or the reference set of 27 most frequent alleles. In step 4, the default setting is for a moderate number of predicted peptides with duplicate peptides removed and the input protein will be automatically split into 15-mers overlapping by 10 amino acids. An overlap of 8 will be used if the “low number of peptides” option is used, and the overlap of 10 if the “high number of peptides” option is chosen. Alternatively, the tool performs the post-processing step of removing largely overlapping peptides from the prediction set by picking the top peptide from the overlapping set of peptides based on percentile rank.

## Sequence-Based B Cell Epitope Predictions

B cell epitope prediction tools can be accessed from the Analysis Resource pull-down menu or the link on the IEDB home page.^4^ On the web page for this tool, users must input either a protein Swiss-Prot ID or a sequence in plain text format and select the method for the prediction. A description of the different methods can be found on the Help tab, including the references and amino acid scales used in these methods. As with the T cell prediction tools, there is also an Example tab that contains several sample cases that can help familiarize users to the input and output formats and a Reference tab that lists the publications that describe the methods.

Historically, physicochemical properties such as hydrophilicity (57), surface accessibility (58), beta-turns (59, 60), and flexibility (61) were correlated with the occurrence of B cell epitopes in proteins. We implemented these four amino acid physicochemical properties-based methods in this tool. In addition, we implemented a semi-empirical method which makes use of physicochemical properties of amino acids and their frequencies of occurrence to predict linear B cell epitopes (62) and a machine-learning based B cell linear epitope prediction method called BepiPred (63). BepiPred is a prediction method that is based on a combination of HMM and Parker’s hydrophilicity and Levitt’s secondary structure scales. For this reason, BepiPred is the default method. With an AUC value of 0.66, its prediction performance is relatively poor, but better than the other methods available on the web page.

**Figure 4:**
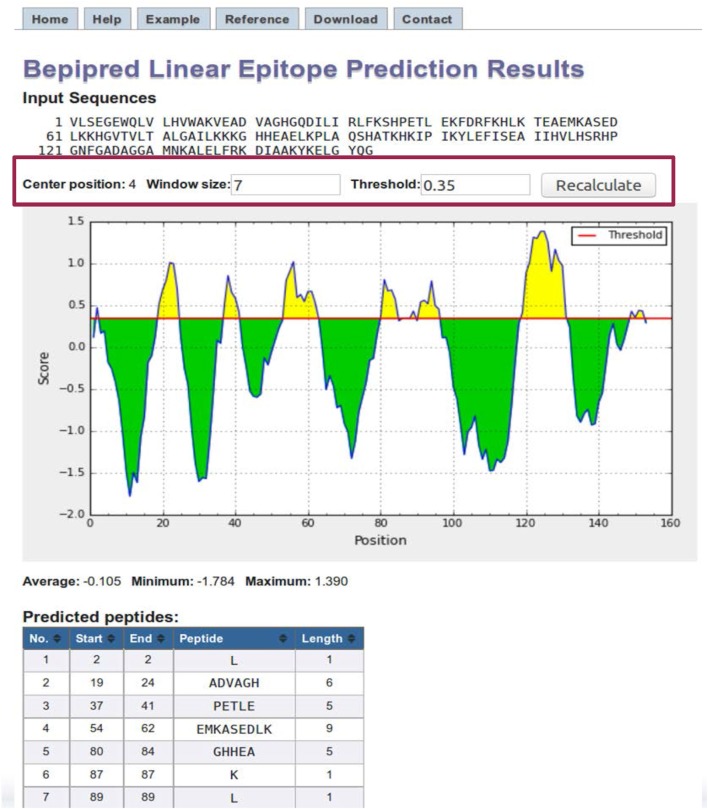
**BepiPred results**. BepiPred results for predicting linear epitopes in sperm whale myoglobin protein (Swissprot ID: P02185). Users can change the window size and score threshold (highlighted in a red box) and recalculate the results. A red line is drawn in the Score versus residue position plot at the chosen score threshold value to predict epitopes. Predicted epitope residue positions are colored in yellow. Predicted peptide table below the plot lists all the predicted linear epitopes and their positions in the protein.

BepiPred results page contains a plot of the predicted score versus the sequence position (Figure 4). The user can adjust the window size and score threshold. The score for a single residue position incorporates the neighboring residues defined by the specified window size, which has a default value of 7. The threshold value is based on the sensitivity and specificity. For BepiPred, a threshold value of 0.35 corresponds to a sensitivity of 0.49 and specificity of 0.75 (63). Increasing the threshold will reduce the sensitivity and increase the specificity, which will reduce false positives but reduce the number of possible epitopes (true positives). A threshold of 0.90 corresponds to a sensitivity of 0.25 and a specificity of 0.91, and a threshold of 1.30 corresponds to a sensitivity of 0.13 and a specificity of 0.96. Below the chart is a table that displays the predicted peptides, start and end positions, sequence, and its length. A second table lists the predicted residue score for each position and an Assignment column that indicates a predicted epitope position with an “E.” For the other methods, the threshold value is automatically set as the average score value, and both the window size and threshold values can be modified by the user.

## Structure-Based Epitope Predictions

The IEDB Analysis Resource has two structure-based methods for predicting discontinuous epitopes—DiscoTope (64, 65) and ElliPro (66). Both make use of the proteins’ geometrical properties. There are also protein–protein docking algorithms that can be used for antigen–antibody interaction prediction. To use structure based prediction tools, the user must provide a three-dimensional (3D) structure of the antigen–antibody complex such as the ones provided by the Protein Data Bank.^5^ The search feature on the home page of the PDB allows the user to search 3D structures of a protein by key work, PDB ID, or a sequence, while an advanced sequence search feature allows one to run BLAST on an input sequence to find relevant structures in the PDB database. Clicking on the desired structure will open a structure specific web page from which the structure coordinates can be downloaded in PDB format for use in the prediction tools. FASTA files are also available for download. PDB ID or a PDB format file is a required input for the DiscoTope epitope prediction method. If a 3D structure is not available in the PDB, there are homology modeling or comparative modeling methods, servers, and databases that can accessed, as described below.

The performance of ElliPro was evaluated in two separate studies in 2007 (67) and 2012 (65). The 2007 benchmark used 42 X-ray structures of antibody–antigen complexes and ElliPro obtained an AUC value of 0.73, a score better than several other predictors, including Epitopia (68), PEPITO (69), and DiscoTope 1 (64). Two antibody–protein docking tools were benchmarked and had AUC values less than 0.60. The 2012 benchmark used 52 X-ray structures of antibody–antigen complexes. In this study, ElliPro had an AUC value of 0.69 and was outperformed by DiscoTope 1.1 (AUC = 0.71) and DiscoTope 2.0 (AUC = 0.73). Overall, the performance of all the structure-based methods has average AUC value less than 0.75. This relatively poor performance might be due to the limited number of structures that can be used to train the algorithms. As more antibody–antigen complexes are deposited in the PDB, the quality of the structure-based predictions may improve.

### The DiscoTope Method for B Cell Epitope Prediction

DiscoTope 2.0 (65) was trained on 75 X-ray structures of antibody–protein complexes and it takes into account multiple epitopes of an antigen. It assigns each residue a score calculated as a linear combination of normalized values from Parker’s hydrophilicity scale, amino acid propensity, the number of contacts within 10 Å for each atom, and the area of relative solvent accessibility. The DiscoTope web page requires three inputs: the PDB ID, a PDB chain ID, and the version of DiscoTope to use. If a user has generated a PDB file from homology modeling, that file can be specified and uploaded as well. The IEDB team recommends the use of DiscoTope 2.0, but it involves a more complex calculation than version 1.1 and subsequently takes longer to run. Users making a large number of runs might, therefore, want to use version 1.1. Different default score thresholds are used by the two different versions.

The initial DiscoTope results page displays a plot of the DiscoTope score versus the amino acid position of the protein, with the score threshold displayed as a red horizontal line and epitope candidate positions colored in green. Users can also select a Table View and a 3D View.

**Figure 5:**
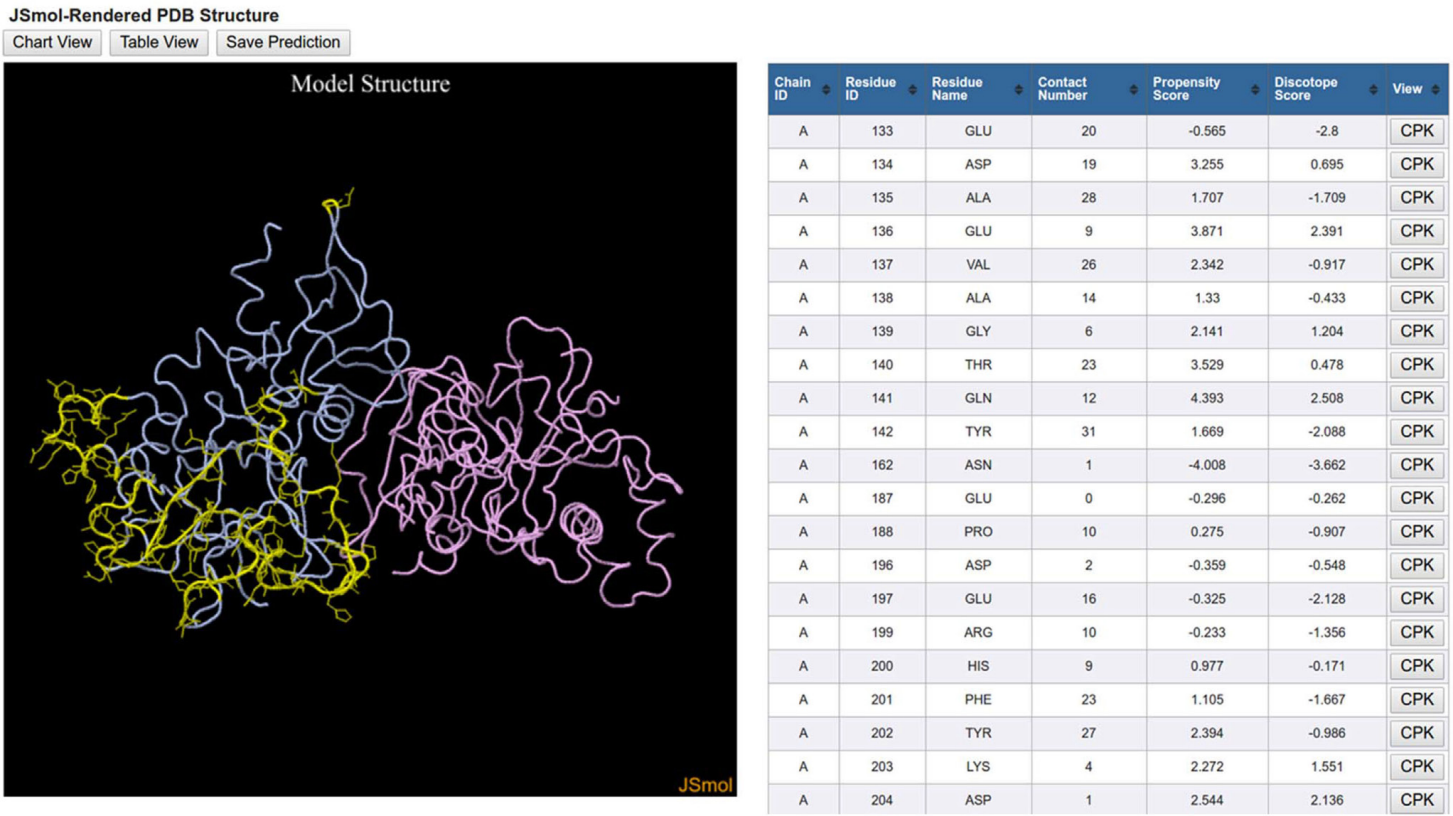
**DiscoTope three-dimensional (3D) view results**. DiscoTope discontinuous epitope prediction results for AMA1 protein from *Plasmodium falciparum* (PDB ID: 1Z40 chain A colored in blue) are shown. 3D structure of AMA1 protein is rendered using JSmol. Predicted epitope residues are shown in yellow. The table on the right side lists all these predicted epitope residues along with different scores calculated by the DiscTope algorithm. Any of these residues can be highlighted in the 3D structure by clicking the CPK button in the table.

The 3D View initiates a JSmol applet that graphically displays the 3D protein structure along with the same table that is displayed in the Table View (Figure 5). The orientation of the 3D protein structure can be changed in JSmol using a computer mouse and visualization settings can be changed by right-clicking the mouse button to reveal a list of display options. Each row in the table has a button labeled CPK, which when clicked will highlight the residue in the structure model.

### ElliPro

Like DiscoTope, ElliPro can be accessed from the IEDB home page from the Analysis Resource pull-down menu or via the link in the right-hand panel. ElliPro (67) predicts epitopes in three steps. It first approximates the protein shape with an ellipsoid. It next calculates a protrusion index (PI) (70) for each and every residue. The PI is determined by constructing an ellipsoid that encompasses as many residues as possible but excludes that particular residue. Once the ellipsoid is constructed, it computes the ratio of the number of residues contained in the ellipsoid to the total number of residues to produce the PI. In the third step, the program clusters neighboring residues based on PI values to predict epitopes. There are two prediction parameters as inputs. The minimum score has a default value of 0.5 and indicates that any residue with a PI greater than 0.5 is considered an epitope candidate. The maximum distance parameter has a default value of 6, which means that only residues within a 6 Å distance will be clustered together within one epitope.

The ElliPro results page contains two tables, the first one for predicted linear epitopes and the second for predicted discontinuous epitopes. The former includes columns for the chain ID, start and end positions of the epitope, the peptide sequence, the number of residues, the ElliPro score, and buttons to view the 3D structure with a JSmol applet. The JSmol rendering of the protein will show the epitope as spheres and the rest of the protein as lines. The ElliPro score is the average value of the PI for all residues involved. The predicted epitopes are presented in descending order based on their scores. The second table has essentially the same information except each residue is listed with its chain ID, amino acid notation, and sequence position. At the bottom of the page is a link so users can view the individual residue scores in a table and a plot of score versus sequence position.

### Homology Modeling for B Cell Epitope Predictions

If the user has an amino acid sequence for their protein of interest but a PDB structure is not available, the user needs to perform homology modeling to generate a PDB file. In this case, we recommend using Protein Model Portal (PMP).^6^ Users can enter their protein sequence in the search field and hit the Search button. PMP will search major protein databases, such as UniProt, Swiss-Prot, and NCBI, and display results of the query. Users can then select a record and obtain a PDB-formatted file. If no models are found, the user can click the Submit button at the bottom of the query result page to submit their target protein sequence to one of PMP’s registered homology modeling services. The subsequent page displays a list of the protein modeling servers along with a brief description of their server policy. Among them MODELLER is one of the prominent homology modeling suites that has gained vast popularity over the years (71). Once users select a server, registers for it, and submit their sequence, they will receive an email informing them that their results are ready to be retrieved.

The I-TASSER server was the number one server for protein structure prediction in community-wide contests for CASP7, CASP8, CASP9, CASP10, and CASP11 (72, 73). It also has a relatively simple user interface and easily understood parameters. However, I-TASSER is very popular and busy, and a protein of 1,000 amino acids could take several days before the job is finished. I-TASSER will provide five predicted homology models by default. Each model has a *C*-score that indicates the level of confidence of the model. I-TASSER also generates a TM score that indicates how close the top ranked structure is to a natural structure. We recommend only considering the models that have *C*-scores greater than −1.50 and using the model with the highest *C*-score if possible, even though a *C*-score meeting this empirical criterion does not necessarily guarantee the reliability of the model chosen. Once the user has obtained the PDB file of their homology model, they can specify the file for upload for their ElliPro run.

### Methods for Modeling and Docking of Antibody and Protein 3D Structures

The B cell page of the Analysis Resource has a link to a web page that provides information on available methods for modeling and docking antibody and protein 3D structures. The first step in the process is taking the antibody and antigen sequences and developing structure models. If a structure exists in the PDB, this step can be skipped. For modeling the antigen, one can use the protein structure modeling process previously described. For antibody modeling, however, there are specific programs available that take advantage of the inherent structure of an antibody. RosettaAntibody (74) and PIGS (75) are two applications that model antibodies. PIGS, or Prediction of ImmunoGlobulin Structure, is easier to use and has been implemented in the Analysis Resource. Once models for the antigen and antibody have been generated, the docking can be modeled with many different protein–protein docking programs including PatchDock (76) and ClusPro (77).

PIGS can be accessed from the Analysis Resource’s B Cell Tools tab. As with the other tools in the Analysis Resource, PIGS has Help, Example, and Reference tabs. Users also have the option of uploading a sequence file with the light and heavy chain information instead. The PIGS results page displays a JSmol rendering of the modeled antibody structure. Each chain has its own color as do the light and heavy chain loops (L1, L2, L3, H1, H2, H3). There is a button at the bottom of the results page to download the structure file. This file can be opened in a text editor where the user can examine the alignment of the target sequence to the canonical antibody template. If the user wants to edit the alignment, they will have to go to the PIGS home website^7^ since this feature has not been implemented in the IEDB.

With structure files for the antibody and the antigen, the user can now use one of the antibody–antigen docking programs. PatchDock^8^ is relatively fast and has good accuracy and a straightforward and easy to understand user interface. The Complex Type input parameter should be set to “Antibody–antigen.” The antibody should be specified as the receptor molecule and the antigen specified as the ligand molecule since the antibody–antigen docking is optimized for this configuration. The Clustering RMSD field is used for clustering the results. Because there can be many results, the program clusters them to be able to present representative ones. PatchDock recommends using 4.0 Å in protein–protein docking. Once a job is submitted, PatchDock typically returns results within a few minutes. The results are ranked by the geometric shape complementarity score (Score), where the higher score indicates a better result. Values are also presented for the approximate interface area of the complex (Area) and the atomic contact energy. The page has a link to visualize the candidate with a Jmol applet and a link to download the results. Users can improve the solutions using the FireDock (78, 79) link at the bottom of the page. PatchDock performs a rigid-body computation so the backbone does not move. FireDock is an efficient method for refining the rigid-body docking solutions of PatchDock.

ClusPro^9^ is another well-known docking server and was the first web-based antibody–antigen docking program publicly available. ClusPro also incorporates electrostatic interaction energy and desolvation energy. ClusPro accepts both PDB IDs and uploaded PDB files as input. Under Advanced Options, there is an Antibody Mode, where the user can check the boxes to use the Antibody Mode and to automatically mask non-CDR regions. The resulting models can be downloaded and visualized on this page.

### Analysis Tools

The IEDB’s analysis tools most frequently used are the population coverage tool, the epitope conservancy tool, and the epitope clustering tool. The population coverage tool (80) calculates the fraction of individuals that are predicted to respond to a given set of epitopes with known MHC restriction, based on HLA genotypic frequencies from http://allelefrequencies.net (81) assuming no linkage disequilibrium between HLA loci. Example data sets from the tab of the same name can be selected. The user can enter either epitope names or sequences, and then selects the population geographical location of interest. Results show what fraction of the population are expected to respond to at least one peptide, how many peptides on average each subject is expected to respond, and to how many peptides 90% of donors can potentially respond. Results are presented in graphical and tabular form.

The epitope conservancy analysis (82) tool calculates the degree of conservancy of an epitope within a given protein sequence set at different degrees of sequence identify. The degree of conservation is defined as the fraction of protein sequences that contain the epitope at a given identity level. In practice, the user inputs a set of epitopes and a set of protein sequences, and specifies the sequence identity threshold. The results page shows the number of protein sequences where each epitope is conserved at the given identity threshold. Users can click on a link to view details for a given epitope, which shows the subsequences in each protein that match the epitope sequence. With an identity threshold of 100%, the matches are identical, but lower thresholds allow varying degrees of amino acid substitutions.

The epitope cluster analysis tool (2) groups epitopes together based on sequence similarity. It is common to have variants of the same epitope tested by different labs that synthesize peptides of different lengths or use different isoforms of proteins. This can result in largely redundant sequences in a group. This tool takes epitope sequences as input and groups them by a user-specified level of identity.

## Conclusion

**Figure 6:**
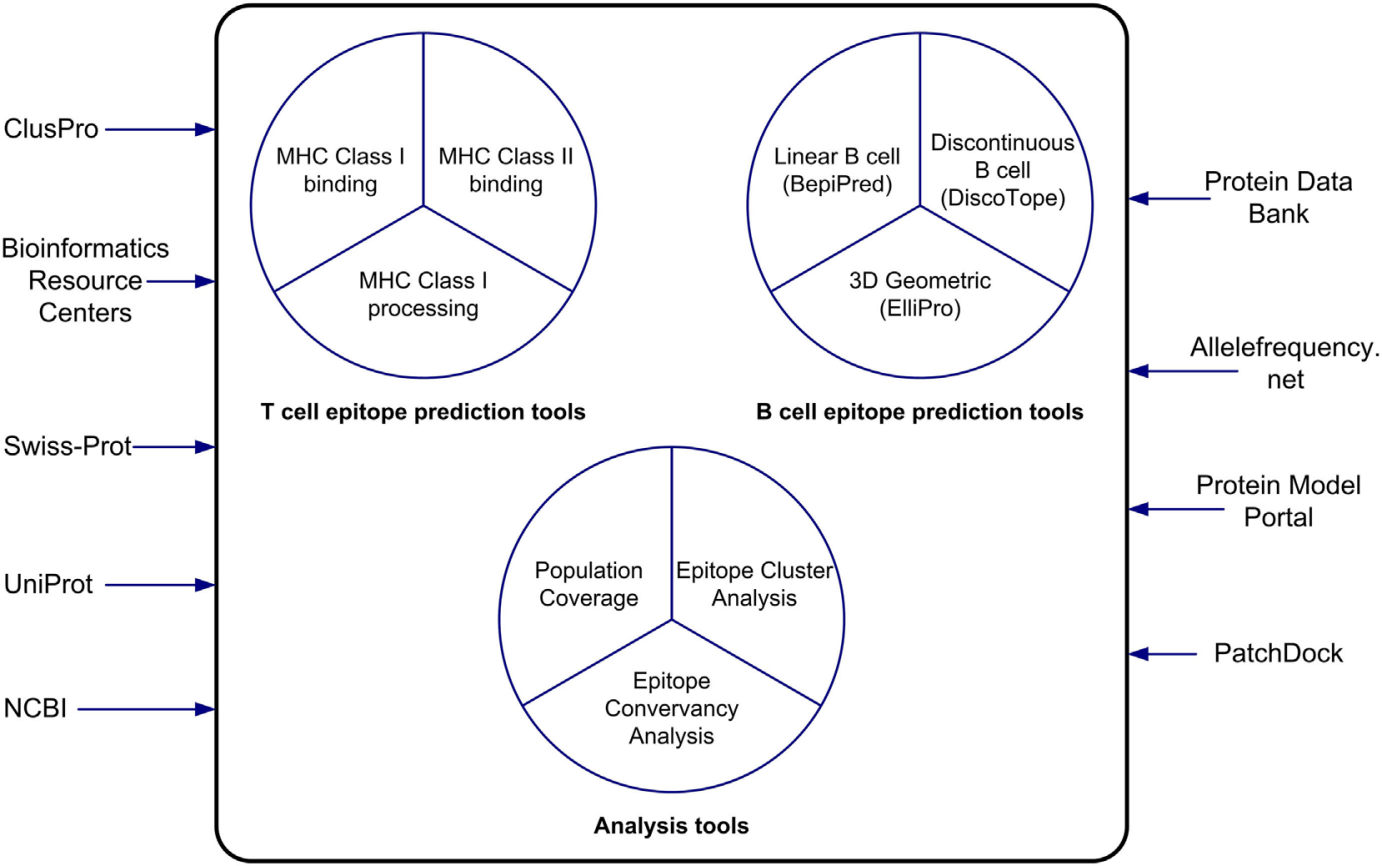
**The tools of the Analysis Resource can be used to predict T cell and B cell epitopes and to analyze sets of epitopes**. The Analysis Resource interacts with a range of bioinformatics resources.

The IEDB and the Analysis Resource provide researchers interested in vaccine design and evaluation with useful bioinformatics resources in the context of data and tools. In this paper, we have described the features of the epitope prediction and analysis tools and how to use them (Figure 6). The MHC class I binding and processing prediction tools calculate putative epitopes and their affinity to a wide assortment of MHC alleles, which correlates to CD8+ recognition and immune response. Evaluations have shown these predictions to have very good accuracy. The MHC class II binding tools, while not as accurate as the class I tools, have improved significantly over the years as more binding data have become available to train the machine learning algorithms that drive the predictions. For both class I and class II binding prediction tools, reference sets of HLA alleles have been derived that provide over 95% global population coverage, an important feature for developing drugs. These tools are helpful in developing a set of likely high affinity binding peptides that can be synthesized for experimental analysis. A user-friendly interface named TepiTool has been developed to guide users through the process of making class I and class II binding predictions. It facilitates the selection of different input parameters, including the IEDB recommended default values and the reference allele sets, and it allows the user to select output criteria, such as the top binders based on percentile rank or promiscuity in the case of class II alleles. The class I immunogenicity tool predicts the immunogenicity of peptide–MHC complex in a manner that is independent of MHC restriction.

The development of B cell epitope predictors is an ongoing area of research. The Analysis Resource offers tools for predicting linear and conformational antibody epitopes using several approaches, including amino acid scales, machine learning techniques, and molecular geometrical properties. Although not providing a performance similar to that of MHC class I and class II predictors, the B cell epitope tools can identify candidate antigen regions likely to bind antibodies. The Analysis Resource also provides a tool for predicting the population coverage of T cell epitope-based vaccines so that vaccines can be designed to maximize coverage. The epitope conservancy analysis tool was designed to analyze the variability and conservation of epitopes within a given set of protein sequences, useful information in developing peptide-based vaccines since conserved epitopes would be expected to be immunogenic across multiple strains or possibly species. In all, the IEDB offers a valuable and free bioinformatics resource to the vaccine design community.

## Author Contributions

WF generated the first draft of the manuscript; SP and SD provided content and figures for the T cell sections; SM provided content and figures for the B cell section, XX provided content for the B cell section; AS and BP edited successive rounds of the manuscript.

## Conflict of Interest Statement

The authors declare that the research was conducted in the absence of any commercial or financial relationships that could be construed as a potential conflict of interest.

## Footnotes

^1^http://tools.iedb.org.

^2^http://tools.iedb.org/main/tools-api/.

^3^http://bio.dfci.harvard.edu/DFRMLI/HTML/natural.php.

^4^http://www.iedb.org.

^5^http://www.rcsb.org.

^6^http://www.proteinmodelportal.org.

^7^http://circe.med.uniroma1.it/pigs/.

^8^http://bioinfo3d.cs.tau.ac.il/PatchDock/.

^9^https://cluspro.bu.edu/.
